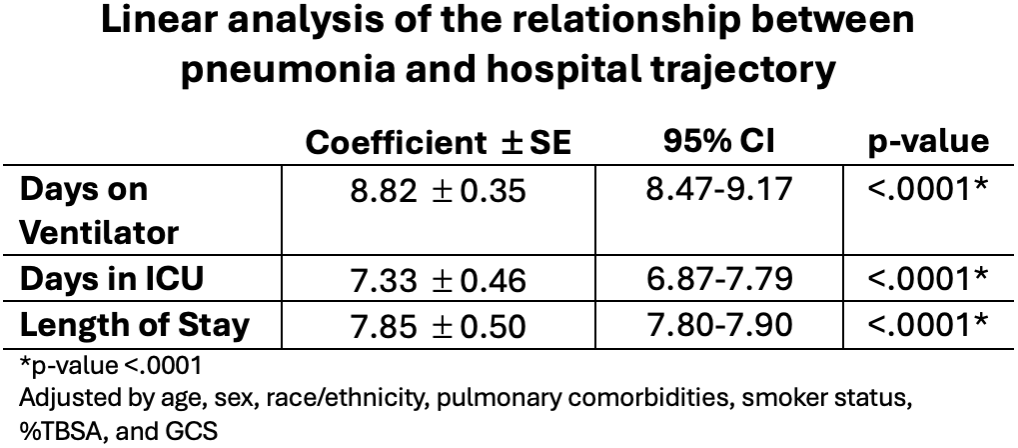# 958 Pneumonia as a Secondary Complication to Inhalation Injury and Its Impact on Hospital Course

**DOI:** 10.1093/jbcr/iraf019.489

**Published:** 2025-04-01

**Authors:** Ryan Johnson, Abigail Plum, John Kubasiak, Manuel Portalatin

**Affiliations:** Loyola University Chicago; Loyola University Chicago; Loyola University Chicago; Loyola University Medical Center

## Abstract

**Introduction:**

Inhalation injury greatly contributes to the morbidity and mortality of burn patients. With limited treatment options for inhalation injury, burn patients are at an increased risk of developing secondary respiratory complications, such as pneumonia (PNA). The impact of secondary respiratory complications on the course of stay for burn patients with inhalation injury remains poorly described. This study aims to assess the association between PNA, developed in the setting of inhalation injury, and hospital outcomes.

**Methods:**

The study utilized the American Burn Association Burn Care Quality Platform (BCQP), consisting of patients ≥18 years of age who were hospitalized for a burn injury between 2013-2022. Inclusion criteria included patients who were reported to have an inhalation injury. Patients were excluded if their mortality outcome was unknown. Pneumonia was queried as any of the ICD-10 diagnoses including pneumonia, aspiration PNA, ventilator-associated PNA, and PNA that is not ventilator-associated. The outcomes of interest included mortality, days on a ventilator, days in the intensive care unit (ICU), and length of hospital stay (LOS). Multivariable linear regression and logistic regression models were conducted to assess the relationship between pneumonia and the outcomes of interest among inhalation injury patients.

**Results:**

Among burn patients with inhalation injuries, more than 1 in 4 patients died during their hospital stay. Mortality was greater for those of older age who were female, non-Hispanic white, non-smokers, with a larger total body surface area burn, and who spent fewer days on the ventilator, in the ICU, and overall LOS. Burn patients complicated by PNA had 64% less odd of mortality compared to those without pneumonia while adjusting for age, sex, race, ethnicity, Glasgow Coma Scale, pulmonary comorbidities, smoker status, and total body surface area (OR=0.36 95% CI 0.34-0.38). Pneumonia complication was found to be significantly associated with an increase of 8.82 +/- 0.35 days spent on a ventilator, 7.33 +/- 0.46 days spent in the intensive care unit, and 7.85 +/- 0.50 additional days in their overall length of stay.

**Conclusions:**

Pneumonia, as a secondary complication to inhalation injury, significantly impacts the patient’s course of hospitalization by increasing the need for mechanical ventilation, ICU treatment, and overall LOS in the hospital. This study emphasizes the need for further improvement in inhalation injury treatment to prevent the onset of secondary complications.

**Applicability of Research to Practice:**

This work identifies the impact of a single pneumonia episode on the hospital trajectory of a patient with inhalation injury. Future work is needed to identify optimal treatment patterns to minimize the risk of pneumonia development.

**Funding for the Study:**

N/A